# A prospective, multi-centre, observational study to examine kidney disease progression in adults with chronic kidney disease – CKDOD - Study design and preliminary results

**DOI:** 10.1186/s12882-015-0191-5

**Published:** 2015-12-22

**Authors:** Bharat Shah, Ashok Kirpalani, Sham Sunder, Ashwani Gupta, Umesh Khanna, Deodatta Chafekar, Li Ping Tan, Dhavee Sirivongs, Dilip Pahari, Gokul Nath, Talat Alp Ikizler

**Affiliations:** Anil Clinic, Mumbai, India; Kidney and blood pressure clinic, Mumbai, India; Department of Nephrology, RML (Ram Manohar Lohiya) hospital, New Delhi, India; Intermed Superspeciality Clinic, New Delhi, India; Lancelot Hospital, Mumbai, India; Supreme Kidney Care, Nashik, India; University Malaya Medical Centre, Renal unit, Faculty of Medicine, Kuala Lumpur, Malaysia; Department of Medicine, Khon Kaen Medical School, Khon Kaen City, Thailand; Dr. Medica Institute of Kidney Diseases, Kolkata, India; Department of Nephrology, St. John’s Medical College & Hospital, Bangalore, India; Vanderbilt University Medical Center, Division of Nephrology, 1161 21st Avenue South, S-3223 Medical Center North, Nashville, TN 37232-2372 USA

**Keywords:** Chronic kidney disease, Clinical registry, Ketoanalogue supplementation

## Abstract

**Background:**

The objective of this article is to describe the organisation of an international, clinical registry, the Chronic Kidney Disease Observational Database (CKDOD), the processes of enrolling patients and entering data and preliminary results to date.

**Design:**

The Chronic Kidney Disease Observational Database (CKDOD) is designed to assess the association between different factors with a known influence on chronic kidney disease (CKD) progression as well as treatment strategies such as dietary modifications, blood pressure control and pharmacological interventions in Asian countries (India, China, Malaysia and Thailand). The only inclusion criterion is the presence of CKD stage 2 or higher as defined by the KDIGO guidelines. Demographic and clinical information are collected by a standardised electronic questionnaire, available in English and Chinese. The data are transferred to the CKDOD database either by e-mail or via web access. All data are checked for consistency and missing values.

Collection of data started in September 2011 and by April 2015, data on 1323 individual patients had been submitted. The mean age at inclusion was 57 ± 14 years, 67 % were male and 36 % were diabetic. The baseline estimated glomerular filtration rate was 26 ml/min/1.73 m^2^. Of all enrolled patients, 324 (24 %) received ketoanalogue supplementation during at least one recorded visit.

**Discussion:**

The CKDOD is a very large and comprehensive data repository, currently focused in subjects recruited from Asia. The database is expected to provide important long-term information on CKD progression, nutritional and metabolic derangements that accompany CKD progression and treatment strategies to ameliorate progression and complications of CKD.

**Trial Registration:**

Clinical Trial Registry – India: CTRI/2012/06/002743; 25th July 2012

**Electronic supplementary material:**

The online version of this article (doi:10.1186/s12882-015-0191-5) contains supplementary material, which is available to authorized users.

## Background

Chronic kidney disease (CKD) is known to be progressive, gradually leading to end-stage renal disease (ESRD) [1]. Incidence and prevalence of CKD are increasing with high burden of morbidity and mortality accounting for 1.4 % of all global deaths in 2010 [2]. Furthermore, CKD patients are far more likely to die from cardiovascular disease before they develop ESRD [3]. Therefore, the actual health and humanistic burden of CKD is underestimated when only death caused by kidney failure is considered. Consequently, CKD, which usually leads to dialysis, has a major impact on public health care and possesses a significant economic and social strain on many countries. India, as well as many developing countries, does not have reimbursement from government for management of ESRD including maintenance dialysis or transplantation. Of the approximately 100,000 Indian patients who develop ESRD each year, 90 % never see a nephrologist. Of those patients who actually start maintenance dialysis, about 60 % are lost to follow up within three months because of the high costs [4].

Loss of kidney function in CKD can be delayed but generally cannot be reversed. Therefore, slowing down progression towards ESRD and amelioration of nutritional and metabolic complications of CKD are the mainstay of CKD management. Especially in countries where renal replacement therapy is not affordable for the majority of the population, a delayed progression to ESRD could significantly increase life expectancy. Factors that influence development and progression of CKD include hypertension, hyperglycaemia, dyslipidaemia, obesity, age, smoking, history of cardiovascular disease and exposure to nephrotoxic agents. Most of these factors also directly influence the nutritional and metabolic status of the CKD patients. The relative contribution of most of the factors towards the progression of CKD have not yet been evaluated in long-term trials, especially in developing countries where most CKD patients are not offered renal replacement therapy [1].

The Chronic Kidney Disease Observational Database (CKDOD) is designed to assess the association between different factors with a known influence on CKD progression as well as treatment strategies such as dietary modifications, keto acid supplementation, blood pressure control and pharmacological interventions in Asian countries. CKDOD aims to document practice patterns and long-term outcomes of CKD patients with the overall goal of providing opportunities for potential treatment strategies. The current manuscript describes the rationale, design and preliminary results of CKDOD.

## Methods

### Organisation and objectives of the registry

CKDOD was initiated by an informal group of Indian nephrologists in 2011 and initially included four centres from India. By 2014, the registry included 12 centres from India, Malaysia, China and Thailand.

The primary objectives of the registry are the evaluation of the current practise patterns for management of CKD patients in Asian countries as well as the analysis of associations between different treatment strategies, including but not limited to nutritional interventions (low protein diet and ketoanalogue supplementation) and blood pressure control and the rate of progression of CKD, incidence of ESRD, transplantation and death. Secondary objectives include assessing the incidence of cardiovascular events, incidence and causes of comorbidities, clinically significant laboratory abnormalities related to metabolic and hormonal derangements as well as quality of life scores.

### Collection of data

All data in the registry are collected following approval by the corresponding ethics committees of the participating centres (See Additional file 1 for SPIRIT Checklist) (Lancelot Independent Ethics Committee, Mumbai, India; Ethics Committee Sir Ganga Ram Hospital, Delhi, India; Shatabdi Hospital Ethics Committee, Nashik, India; Clinical Research Ethics Committee of the MEDICA Superspecialty Hospital, Kolkata, India; St. John’s Medical College & Hospiutal Institutional Ethical Review Board, Bangalore, India; Institutional Ethics Committee of the Dr. Tam Manohar Lohia Hospital, New Delhi, India; Medical Ethics Committee of the University Malaya Medical Centre, Kuala Lumpur, Malaysia; Khon Kaen University Ethics Committee for Human Research, Khon Kaen, Thailand; Medical Ethics Committee of the Qilu Hospital of Shandong University, Shandong, China).

The main inclusion criterion for the registry is the presence of CKD stages 2 to 5 not on dialysis, defined by an estimated glomerular filtration rate (eGFR) below 90 ml/min per 1.73 m^2^. Other inclusion criteria include individuals aged at least 18 years, with a regular attendance at a given clinical site, a life expectancy of at least six months and written informed consent. The responsibility of obtaining and storing written informed consent lies with the participating centres. Patients are excluded if they have or have had a kidney transplant, sustained sitting systolic blood pressure (BP) > 160 mmHg and sitting diastolic BP > 110 mmHg despite appropriate therapy or an independent life-threatening disease such as active cancer, HIV or end-stage liver or heart disease.

Each participating physician or his/her staff transfers the data from source documents (medical records) to the secured (username/password protected) CKDOD website (online data collection tool) or the offline data collection tool. The offline version is used for sites where internet is inaccessible or connection is unstable. Data input to the website updates the database in real time. Data input to the offline data collection tool is transferred to the online database once internet connection becomes available.

Each centre is provided with a unique login ID (user name) and password. The user can only see the patients entered by his centre. The identity of the patient is not visible in the web application.

Data analysis is done after patient de-identification for the data coordinating site. Anonymity of the data is assured by coding the included patients with an identification number consisting of a four-digit field automatically generated by the web application. Once assigned to a patient, the patient number is not replaced or reused. The actual patient name is only known to the participating centre and is not saved in the database. Only the centres keep lists with the names of the patients and the respective identification number.

The data management team performs consistency checks and issues electronic data clarification forms to follow up on discrepant data. Data exceeding plausible ranges or missing data are identified by the data acquisition programme during data entry. A quarterly manual review is done to check consistency and completeness of data. Study sites receive monthly reports on data quality and completeness in order to verify, correct, or complete irregular or missing data. If data modification is required, changes on the CKDOD website database are only conducted after confirmation from the source documents by the responsible investigator or study nurse.

**Fig. 1:**
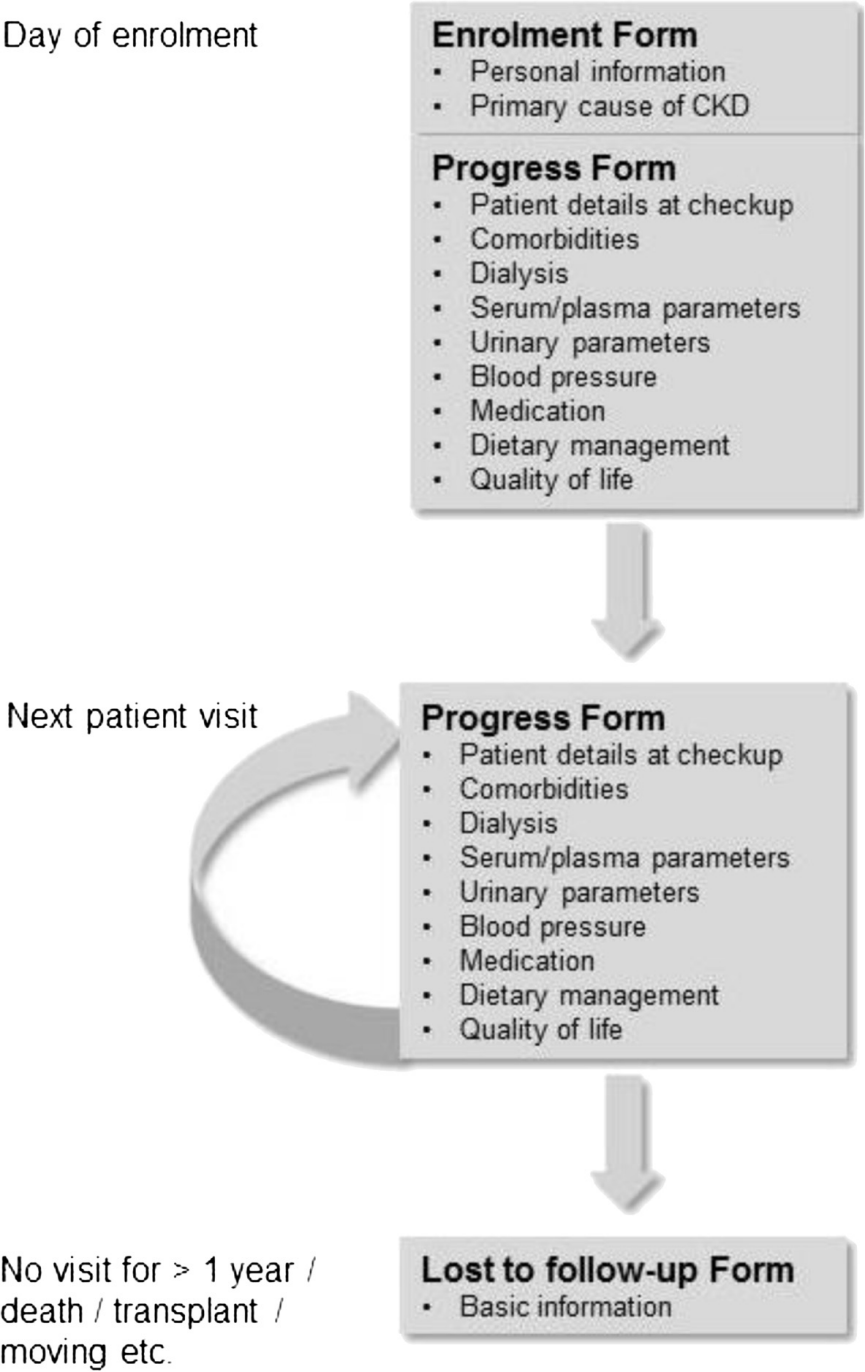
Documentation procedure

This study does not require any study specific treatments or investigations. Patients are treated and managed according to the physicians’ clinical judgment. During the study period, data are recorded from assessments and evaluations performed according to routine practice and standard care at the centres and subsequently entered into a web-based data portal. The data for this study are collected for each patient until death, renal transplant, and withdrawal of consent or loss to follow up due to any other reason. Data collected at enrollment includes baseline demographic data and presumed cause of CKD. The questionnaire is completed at each visit, including the day of enrollment, includes data on serum and urinary parameters, dialysis, comorbidities, medications, nutritional interventions and quality of life (Additional file 2). Patients who have no visit record for more than one year or have died, received a renal transplant or were transferred to a different centre are considered as lost to follow up. Figure 1 depicts the documentation procedure.

During the follow up period, no diagnostic or monitoring procedures additional to standard care and routine practice are applied. Only data originating from assessments and evaluations performed according to the centres’ routine practice are recorded for the purpose of this study. Patient visit intervals are chosen at the physician’s discretion and according to their standard of care.

### Analysis

A first preliminary and solely descriptive analysis has been performed for this particular report. For all parameters shown in Table 2, the mean and standard deviation has been calculated for the total population and for each country separately.

After the end of the documentation period, correlations between different factors influencing the progression of CKD as well as treatment strategies and the rate of progression towards ESRD will be calculated. Primary endpoints are progression of CKD defined by reaching CKD stage 5 and decline of GFR during the study period. Standard analyses will be carried out to describe patients and clinical practices, overall and by subgroups defined by dietary protein and energy intake, as well as keto acid supplementation. Multivariate regression models will be used to study the associations between patient characteristics, clinical practices, or nutritional interventions and the progression of CKD. We calculated the required sample size assuming 20 % of patients receiving keto acid treatment and based on published data [5]. Targeting a power of 0.9 the required sample size is about 700 patients. For detection of differences of GFR decline this number would also be sufficient expecting data similar to previously published data [6]. Taking into account possible drop out and less defined conditions as in the two RCTs we aim at including at least 1,500 patients to the registry.

## Results

**Fig. 2:**
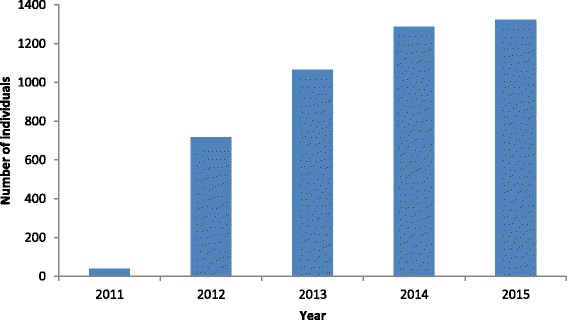
Cumulative increase of patients included into the CKDOD registry. By April 2015, a total of 1323 individuals were included in the registry

The number of patients for whom data has been submitted is shown in Fig. 2. Collection of data began in September 2011 and by April 2015, data from 1323 individuals has been submitted. Table 1 shows the number of subjects by country and the year when each country included its first patient (updated April 2015). The mean age of the subjects was 57 years (range 18–102 years; SD 14 years) and 67 % were male. Additional baseline data is shown in Table 2.

**Table 1:** The number of patients included in the CKDOD registry by country. Last updated in April 2015

Country	Subjects (N)	First patient included (year)
Total	1323	2011
India	1137	2011
Thailand	56	2012
Malaysia	80	2013
China	50	2014

**Table 2:** Demographic data and baseline information

	Total	India	Thailand	Malaysia	China
Number of patients enrolled, N	1323	1137	56	80	50
Males, %	881 (67 %)	759 (67 %)	36 (64 %)	53 (66 %)	33 (66 %)
Age, mean (SD)	57 (±14)	56 (±14)	67 (±12)	68 (±10)	50 (±15)
BMI, mean (SD)	25.2 (±4.8)	25.2 (±4.8)	24.1 (±4.8)	26.8 (±5.7)	24.6 (±3.4)
First blood pressure in mmHg, mean (SD)	135/81 (±15/10)	134/83 (±15/9)	133/68 (±17/11)	139/74 (±15/13)	132/82 (±13/11)
First creatinine in mg/dL, mean (SD)	3.2 (±1.9)	3.4 (±2.0)	2.6 (±1.2)	2.4 (±0.6)	2.1 (±1.3)
First eGFR in ml/min/1.73 m2 (EPI), mean (SD)	26.0 (±16.4	25.0 (±16.2)	26.8 (±12.2)	26.8 (±8.4)	44.8 (±23.0)
Patients on low or very low protein diet with ketoanalogue supplementation at any visit, %	324 (24 %)	284 (25 %)	2 (4 %)	20 (25 %)	18 (36 %)
Patients on low or very low protein diet without ketoanalogue supplementation at any visit, %	249 (19 %)	178 (16 %)	4 (7 %)	37 (46 %)	30 (60 %)

**Fig. 3:**
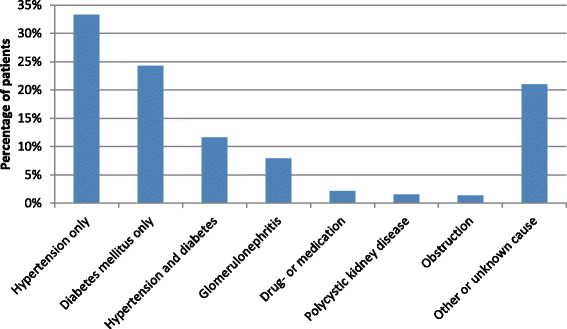
Primary cause of CKD. More than one cause could be selected

The most often entered primary cause of CKD was hypertension, followed by diabetes mellitus (33 % of the patients had only hypertension, 24 % only diabetes, and 12 % both hypertension and diabetes, Fig. 3). At the first recorded visit, the majority of patients had CKD stage 4 (35 %), followed by stage 3 (27 %), and stage 5 (26 %).

Of all enrolled patients, 237 (17,9 %) were vegetarians. 228 (17,2 %) consulted a dietician at least at one visit and 194 (14,7 %) were using a nutrition diary. The average recommended caloric intake at the first visit was 29,0 kcal/kg body weight daily. The estimated actual caloric intake was 28,8 kcal/kg. 375 (28 %) were prescribed a low or very low diet. Of these, 324 (24 %) received ketoanalogue supplementation at least once. The usual dose was six tablets per day. 249 (19 %) of the patients were on a low or very low protein diet without ketoanalogue supplementation (Table 2).

## Discussion

In the present paper, we describe the design of Chronic Kidney Disease Observational Database, an international pragmatic cohort study aimed at providing information on practice patterns of CKD management in developing countries with limited access to renal replacement therapies.

The worldwide increase in prevalence of CKD and the enormous costs of treatment are already a major burden in developed countries, but the impact of CKD on developing countries is far more challenging. The clinical, epidemiologic, and socioeconomic effects of CKD are expected to be greatest in low- or middle-income countries, which comprise about 85 % of the world’s population. By the year 2030, an estimated 70 % of patients with ESRD will be residents of these countries [7]. Due to the absence of any registry, the actual incidence of ESRD can only be estimated for India or other southern Asian countries [8]. In addition to the treatment of patients with ESRD the organisational and financial resources needed for the prevention and detection of CKD pose a major problem for low- or middle-income countries.

Besides missing information on incidence and prevalence of CKD and ESRD, little is known about the actual treatment of patients in India and other Asian countries. A large Asian registry including patients treated at numerous nephrology centres offers the opportunity to assess current treatment practices and analyse the effects of different treatment strategies on the progression of CKD with the aim of improving treatment practice in the long run.

With 1323 subjects enrolled (last updated April 2015), CKDOD is a large and comprehensive registry for monitoring the progression of CKD in Asia. This registry is to our knowledge the first of its kind to combine information on CKD progression, treatment and nutritional intervention in a long-term data collection. A search at ClinicalTrials.gov revealed 566 observational studies with the term “chronic kidney disease” and 46 studies when the term “diet” was added. However, to our knowledge no other observational study focuses on the interactions between the type of protein restriction (low protein diet), the supplementation with ketoanalogues and the progression of CKD, which might be a very important especially in low resource settings for mangement of advanced CKD. In Germany, a large chronic kidney disease registry with more than 6000 included patients is expected to reveal interesting information about clinical treatment variation and the impact of clinical treatments on survival, quality of life and cost [9]. However, to our knowledge this registry does not consider information on diet and nutritional supplementation. The Chronic Renal Insufficiency Cohort (CRIC) study started in the US [10] uses a detailed food questionnaire to evaluate possible effects of nutritional habits but does not focus on protein restriction and ketoanalogue supplementation. The Chronic Kidney Disease Japan Cohort (CKD-JAC) study focuses on correlation of CKD and cardiovascular disease. [11] The Korean KNOW-CKD study [12] does not seem to focus on nutritional aspects. Finally, the French Chronic Kidney Disease-Renal Epidemiology and Information Network (CKD-REIN) has a strong focus on pharmacoeconomic aspects but also not on nutrition.[13] The Indian Clinical Database of Kidney Diseases (CDKD) collects kidney-related physiological data [14] but no information on diet of CKD patients and is no longer maintained. The United States Renal Data System (USRDS) may be the largest data collection on CKD, but provides mainly data on incidence and prevalence and not on progression or nutritional management.

Running CKDOD with a large Indian population also provides us with the opportunity to study the progression of CKD under ketoanalogue supplementation in patients with a habitually low protein intake. Patel et al. showed that even without protein restriction, protein intake was 0.65 g/kg/day in Indian CKD patients [15], which is very close to a low protein diet. CKDOD will not only result in valuable information about a treatment approach which relies highly on patient compliance and cooperation, but also provide the opportunity of comparing clinical courses and outcomes of different ethnic groups with widely differing diets. The development of a shared questionnaire, the adoption of minimum requirements to ensure quality control and the electronic transfer of data, either by e-mail or by a secure web-enabled database, greatly contributes to the success of the CKDOD data validation, dissemination and rapid growth.

In a recent publication, Aparicio et al. [16] showed that a ketoanalogue-supplemented very low protein diet (sVLPD) can postpone dialysis treatment. Furthermore, Garneata and Mircescu reported that sVLPD can effectively ameliorate metabolic disturbances of advanced CKD and delay the initiation of dialysis without deleterious effects on nutritional status. Piccoli et al. [17] found promising results in terms of mortality and CKD progression for pre-dialysis patients with a supplemented low protein diet. The CKDOD registry is expected to add valuable long-term pragmatic data of a large patient population and shed further light on the possibility to delay dialysis with the right combination of diet and supplementation.

In summary, CKDOD is expected to reveal important information regarding the practice patterns for the care of CKD patients with a specific emphasis on nutritional and metabolic aspects. These data could generate hypotheses for future research or clinical projects in the management of CKD patients.

## Additional files

Additional file 1:
**SPIRIT Checklist for the CKDOD.** Items 11, 17, 22, 30 and 33 were not applicable due to the observational nature of the study and lack of any biospecimen collection. (DOCX 22 kb) Additional file 2:
**Chronic Kidney Disease Observational Database subject data collection forms.** The information was collected by the research staff or the study investigators and subsequently transferred to the secured (username/password protected) CKDOD website (online data collection tool) or the offline data collection tool. The offline version is used for sites where internet is inaccessible or connection is unsecure. (PDF 141 kb)

## Abbreviations

CDKD: Clinical Database of Kidney Diseases
CKD: Chronic kidney disease
CKDOD: Chronic Kidney Disease Observational Database
ESRD: End-stage renal disease
