# Supplementary material for: A prospective, multi-centre, observational study to examine kidney disease progression in adults with chronic kidney disease – CKDOD - Study design and preliminary results
Source: BMC Nephrol. 2015 Dec 22;16:215. doi: 10.1186/s12882-015-0191-5 (PMC4687357; doi:10.1186/s12882-015-0191-5)
Supplement: Additional file 1: — SPIRIT Checklist for the CKDOD. Items 11, 17, 22, 30 and 33 were not applicable due to the observational nature of the study and lack of any biospecimen collection. (DOCX 22 kb) [file 12882_2015_191_MOESM1_ESM.docx]

**SPIRIT Checklist for CKDOD**

| **1** | Title | + |
| --- | --- | --- |
| **2** | Trial registration | + |
| **3** | Protocol | + |
| **4** | Funding | + |
| **5** | Roles and responsibilities | + |
| **6** | Background and rationale | + |
| **7** | Objectives | + |
| **8** | Trial design | + |
| **9** | Study setting | + |
| **10** | Eligibility criteria | + |
| **11** | Interventions | - CKDOD is a non-interventional study |
| **12** | Outcomes | + |
| **13** | Participant timeline | (+) CKDOD is a non-interventional study collecting data from regular clinical routine |
| **14** | Sample size | + |
| **15** | Recruitment | (+) CKDOD is a non-interventional study collecting data from regular clinical routine |
| **16** | Sequence generation | - CKDOD is a non-interventional study |
| **17** | Blinding | - CKDOD is a non-blinded non-interventional study |
| **18** | Data collection methods | + |
| **19** | Data management | + |
| **20** | Statistical methods | + |
| **21** | Monitoring | (+) CKDOD is a non-interventional study collecting data from regular clinical routine |
| **22** | Harms | - CKDOD is a non-interventional study collecting data from regular clinical routine / registry for hypothesis generation |
| **23** | Auditing | + |
| **24** | Research ethics approval | + |
| **25** | Protocol amendments | + |
| **26** | Consent or assent | + |
| **27** | Confidentiality | + |
| **28** | Declaration of interests | + |
| **29** | Access to data | + |
| **30** | Ancillary and post-trial care | - CKDOD is a non-interventional study collecting data from regular clinical routine |
| **31** | Dissemination policy | + |
| **32** | Informed consent materials | + |
| **33** | Biological specimens | - CKDOD is a non-interventional study collecting data from regular clinical routine |
