# Supplementary material for: A prospective, multi-centre, observational study to examine kidney disease progression in adults with chronic kidney disease – CKDOD - Study design and preliminary results
Source: BMC Nephrol. 2015 Dec 22;16:215. doi: 10.1186/s12882-015-0191-5 (PMC4687357; doi:10.1186/s12882-015-0191-5)
Supplement: Additional file 2: — Chronic Kidney Disease Observational Database subject data collection forms. The information was collected by the research staff or the study investigators and subsequently transferred to the secured (username/password protected) CKDOD website (online data collection tool) or the offline data collection tool. The offline version is used for sites where internet is inaccessible or connection is unsecure. (PDF 141 kb) [file 12882_2015_191_MOESM2_ESM.pdf]

Patient enrolment details – CKDOD registry

CKDOD
CHRONIC KIDNEY DISEASE OBSERVATIONAL DATABASE

Patient ID

Centre name

CRC's name

Other

Personal Information

Date of enrolment	D D . M M . Y Y Y Y
Date of birth	D D . M M . Y Y Y Y
Gender	
Descent	
Height	☐ cm ☐ m
History of smoking	Y N U
Date of first diagnosis of CKD	D D . M M . Y Y Y Y

Primary Cause of CKD

- ☐ Diabetes mellitus
- ☐ Hypertension
- ☐ Glomerulonephritis
- ☐ Inherited disease (e.g. polycystic kidney disease)
- ☐ Malformations / birth defects
- ☐ Autoimmune disorder (e.g. lupus)
- ☐ Obstructions (kidney stones, tumours, enlarged prostate gland)
- ☐ Infections
- ☐ Toxic chemicals
- ☐ Injury or trauma
- ☐ Reflux nephropathy
- ☐ Drug- or medication-induced kidney problems
- ☐ Other
- ☐ Unknown

Date and signature

Y=Yes N=No U=Unknown

CHRONIC KIDNEY DISEASE OBSERVATIONAL DATABASE

Progress form – CKDOD registry

Patient ID

Centre name

CRC's name

Other

General Information

Date of visit D D . M M . Y Y Y Y

Patient Details at Checkup

Is the patient currently on dialysis?	Y N U
Body weight	☐ kg ☐ lb
Waist circumference	cm
Is the patient pregnant?	Y N U
CKD-related hospitalisation since last visit?	Y N U
Primary diagnosis for hospitalisation	
Secondary diagnosis for hospitalisation	

Comorbidities

Chronic metabolic acidosis (serum bicarbonate level below 22 mmol/L)	Y N U
AIDS (with clinical symptoms besides being HIV positive)	Y N U
Osteoporosis (only if patient is treated for osteoporosis)	Y N U
Denture (if yes, is it fitted or not?)	
Periodontal disease	Y N U
Connective tissue disease (e.g. rheumatoid arthritis)	Y N U
Cerebrovascular disease (stroke with mild or no residual or TIA)	Y N U
Dementia	Y N U
Hemiplegia (severe weakness of arm/leg on one side of the body)	Y N U
Myocardial infarction (clinical event, not ECG changes only)	Y N U
Congestive heart failure (inability of the heart for sufficient pump action)	Y N U
Peripheral vascular disease (includes aortic aneurysm >= 6 cm)	Y N U
Chronic pulmonary disease	Y N U
Peptic ulcer disease	Y N U

Y=Yes N=No U=Unknown

CKDOD
CHRONIC KIDNEY DISEASE OBSERVATIONAL DATABASE

Progress form – CKDOD registry

Patient ID	Centre name	CRC's name

Other:

	Y	N	U
Mild liver disease (without portal hypertension, includes chronic hepatitis)	Y	N	U
Moderate or severe liver disease	Y	N	U
Diabetes without end-organ damage (excludes diet-controlled alone)	Y	N	U
Diabetes with end-organ damage (retinopathy, neuropathy, nephropathy, or or brittle diabetes)	Y	N	U
Nephrotic syndrome	Y	N	U
Moderate to severe renal disease (CKD stage 3 to 5)	Y	N	U
Tumour without metastasis (check "no" if more than 5 y since diagnosis)	Y	N	U
Metastatic solid tumour	Y	N	U
Leukaemia (acute or chronic)	Y	N	U
Lymphoma	Y	N	U
Inflammation	Y	N	U
Muscle-wasting disease	Y	N	U

Dialysis

Type of dialysis (none, HD, HDF, PD)	
Dialysis frequency per week	
When did dialysis start?	D D . M M . Y Y Y Y
What type of dialyser is used?	

Serum / Plasma Parameters

		Units
Creatinine		☐ mg/dL ☐ µmol/L
BUN (blood urea nitrogen)		☐ mg/dL ☐ mmol/L
Sodium		mmol/L
Potassium		☐ mEq/L ☐ mmol/L
Total protein		☐ g/dL ☐ g/L
Bicarbonate		☐ mEq/L ☐ mmol/L
Uric acid		☐ mg/dL ☐ µmol/L

Y=Yes N=No U=Unknown

CKDOD
CHRONIC KIDNEY DISEASE OBSERVATIONAL DATABASE

Progress form – CKDOD registry

Patient ID	Centre name	CRC's name

Other

Calcium		☐ mg/dL ☐ mEq/L ☐ mmol/L
Phosphate		☐ mg/dL ☐ mmol/L
Hemoglobin		☐ g/dL ☐ g/L ☐ mmol/L
CRP		mg/L
Total cholesterol		☐ mg/L ☐ mmol/L
LDL cholesterol		☐ mg/dL ☐ mmol/L
HDL cholesterol		☐ mg/dL ☐ mmol/L
Triglycerides		☐ mg/dL ☐ mmol/L
ALT (alanine aminotransferase)		U/L
Urea		☐ mg/dL ☐ mmol/L
Fasting blood glucose		☐ mg/dL ☐ mmol/L
HbA1c		%
pH		
Albumin		g/dL

Urinary Parameters

Urine volume (in 24h)		☐ mL ☐ L
Urea nitrogen (in 24h urine)		☐ g/24h ☐ mmol/24h
Creatinine (in 24h urine)		☐ mg/24h ☐ mmol/24h
Urea (in 24h urine)		☐ mg/24h ☐ mg/min ☐ g/24h ☐ mol/24h
Albumin (in 24h urine)		☐ mg/24h ☐ mg/mmol ☐ mg
Urea nitrogen (in spot urine)		☐ mg/dL ☐ mmol/L
Protein (in spot urine)		☐ mg/dL
Creatinine (in spot urine)		☐ mg/dL
Albumin (in spot urine)		☐ mg/dL ☐ mg/mg urine-creatinine
Proteinuria	Y N U	

Y=Yes N=No U=Unknown

CKDOD
CHRONIC KIDNEY DISEASE OBSERVATIONAL DATABASE

Progress form – CKDOD registry

Patient ID

Centre name

CRC's name

Other

Blood Pressure

Systolic blood pressure mm Hg

Diastolic blood pressure mm Hg

Where / how was it measured? ☐ Centre ☐ At home ☐ Continuoulsy over 24 h

Medication

Sulphonylurea compounds	Y	N	U
Thiazolidinediones / Glitazones	Y	N	U
Alphaglucosidaseinhibitor	Y	N	U
Biguanide	Y	N	U
Dipeptidyl peptidase IV inhibitors	Y	N	U
Meglitinides	Y	N	U
Insulin	Y	N	U
SGLT2 inhibitor (sodium glucose transport protein inhibitor)	Y	N	U
Beta blockers	Y	N	U
ACEI (angiotensin converting enzyme inhibitors)	Y	N	U
Alpha receptor blockers	Y	N	U
Calcium antagonists	Y	N	U
ARB (angiotensin II receptor blockers)	Y	N	U
Loop diuretics	Y	N	U
Potassium sparing diuretics	Y	N	U
Thiazides	Y	N	U
Carbonic anhydrase inhibitors	Y	N	U
DRI (dopamine reuptake inhibitor)	Y	N	U
Phosphate binders	Y	N	U
Allopurinol	Y	N	U
Erythropoitein	Y	N	U
TCM (traditional Chinese medicine)	Y	N	U

Other drugs

Y=Yes N=No U=Unknown

CKDOD
CHRONIC KIDNEY DISEASE OBSERVATIONAL DATABASE

Progress form – CKDOD registry

Patient ID

Centre name

CRC's name

Other

Dietary Management

Ketoanalogues (please specify)

Ketoanalogues dosage tablets/day

Number of ketoanalogue pills prescribed

Date of last ketoanalogue pill prescription

How many ketoanalogue pills does the patient still possess?

If keto supplementation has been discontinued, please select the main reason:

- ☐ Further decline of eGFR
- ☐ Lack of compliance
- ☐ Shift to RRT

PEW (protein energy wasting) suspected? Y N U

Type of protein restriction

- ☐ None
- ☐ VLPD (0.3 - 0.5 g/kg bw/d)
- ☐ LPD (0.6 - 0.8 g/kg bw/d)
- ☐ HPD (greater than 1.0 g/kg bw/d)

Vegetarian? Y N U

Followed up by a dietician? Y N U

Using a nutritionary diary? Y N U

How do you judge the patient's dietary compliance? (1=perfect; 5=not compliant)

Daily amount of consumed calories ☐ g/kg bw/day ☐ g/day

Daily recommended calories (without ketoanalogues) ☐ g/kg bw/day ☐ g/day

Daily amount of consumed protein ☐ g/kg bw/day ☐ g/day

Daily recommended protein (without ketoanalogues) ☐ g/kg bw/day ☐ g/day

Date and signature

CKDOD
CHRONIC KIDNEY DISEASE OBSERVATIONAL DATABASE

Nutritional risk screening – CKDOD registry

Patient ID

Centre name

CRC's name

Other

Please use this form if PEW (protein energy wasting) is suspected

Date of screening D D . M M . Y Y Y Y

Table 1: Initial screening

1. Is BMI below 20.5? Y N
2. Has the patient lost weight within the last 3 months? Y N
3. Has the patient had a reduced dietary intake in the last week? Y N
4. Is the patient severely ill? (e.g. in intensive therapy) Y N

If the answer is 'Yes' to any question, please perform the screening in Table 2.
If the answer is 'No' to all questions, the screening is finished.

Table 2: Final screening (please rate the impaired nutritional status and severity of disease and add both scores)

Impaired nutritional status		Severity of disease (≈ increase in requirements)	
• Normal nutritional status	Absent **Score 0**	• Normal nutritional requirements	Absent **Score 0**
• Weight loss more than 5% in 3 months and/or • Food intake below 50 – 75% of normal requirement in preceding week	Mild **Score 1**	• Hip fracture and/or • Chronic patients, in particular with acute complications: cirrhosis, COPD and/or • Chronic hemodialysis, diabetes, oncology	Mild **Score 1**
• Weight loss more than 5% in 2 months and/or • BMI 18.5 - 20.5 + impaired general condition and/or • Food intake 25 – 50% of normal requirement in preceding week	Moderate **Score 2**	• Major abdominal surgery and/or • Stroke and/or • Severe pneumonia, hematologic malignancy	Moderate **Score 2**
• Weight loss more than 5% in 1 month (more than 15% in 3 months) and/or • BMI below 18.5 + impaired general condition and/or • Food intake 0 – 25% of normal requirement in preceding week	Severe **Score 3**	• Head injury and/or • Bone marrow transplantation and/or • Intensive care patients (APACHE > 10)	Severe **Score 3**
	**Score**		**Score**
		**= Total score:**	
**Age:** if 70 years or older, add 1 to total score above		**= Age-adjusted score:**	

Score 3 or higher: The patient is nutritionally at risk and a nutritional care plan is initiated
Score below 3: Weekly re-screening of the patient. If the patient e.g. is scheduled for a major operation, a preventive nutritional care plan is considered to avoid the associated risk status.

Modified from Kondrup et al. Clinical Nutrition 2003; 22: 415-421

Date and signature

Y=Yes N=No

Quality of life form – CKDOD registry

Patient ID

Centre name

CRC's name

Other

Quality of Life (please refer to the COOP-Charts for details)
All questions refer to the past 4 weeks.

Hardest physical activity possible for at least 2 minutes
- ☐ Very heavy (1)
- ☐ Heavy (2)
- ☐ Moderate (3)
- ☐ Light (4)
- ☐ Very light (5)

Emotional problems
- ☐ Not at all (1)
- ☐ Slightly (2)
- ☐ Moderately (3)
- ☐ Quite a bit (4)
- ☐ Extremely (5)

Difficulty with usual activities
- ☐ No difficulty at all (1)
- ☐ A little bit of difficulty (2)
- ☐ Some difficulty (3)
- ☐ Much difficulty (4)
- ☐ Could not do (5)

Limitations of social activities
- ☐ No (1)
- ☐ Yes, slightly (2)
- ☐ Yes, moderately (3)
- ☐ Yes, a lot (4)
- ☐ Yes, extremely (5)

Pain
- ☐ No pain (1)
- ☐ Very mild pain (2)
- ☐ Mild pain (3)
- ☐ Moderate pain (4)
- ☐ Severe pain (5)

Change in health compared to 4 weeks ago
- ☐ Much better (1)
- ☐ A little better (2)
- ☐ About the same (3)
- ☐ A little worse (4)
- ☐ Much worse (5)

Overall health
- ☐ Excellent (1)
- ☐ Very good (2)
- ☐ Good (3)
- ☐ Fair (4)
- ☐ Poor (5)

Social support
- ☐ Yes, as much as patient wanted (1)
- ☐ Yes, quite a bit (2)
- ☐ Yes, some (3)
- ☐ Yes, a little (4)
- ☐ No, not at all (5)

Quality of life
- ☐ Very well (1)
- ☐ Pretty good (2)
- ☐ Good & bad parts about equal (3)
- ☐ Pretty bad (4)
- ☐ Very bad (5)

Date and signature

CKDOD
CHRONIC KIDNEY DISEASE OBSERVATIONAL DATABASE

Loss to follow-up form – CKDOD registry

Patient ID | Centre name | CRC's name

Other

General Information

Date of recording: D D . M M . Y Y Y Y

Reason for loss to follow-up
- ☐ Patient received kidney transplantation
- ☐ Patient moved
- ☐ Transferred to a colleague
- ☐ Patient died
- ☐ Complications
- ☐ No information

If the patient died, please specify cause
- ☐ Cardiac disease
- ☐ Cerebrovascular disease
- ☐ Infection
- ☐ Malignancy
- ☐ Renal failure
- ☐ Other
- ☐ Unknown

Date of death: D D . M M . Y Y Y Y

If patient was lost to follow-up due to complications, please specify

Date and signature
